# Cardiac contractility modulation to enhance optimized medical therapy and improve cardiac remodeling in advanced heart failure: a case report

**DOI:** 10.3389/fcvm.2025.1577680

**Published:** 2025-06-06

**Authors:** Lina Feng, Lina Su, Jingyi Ren

**Affiliations:** ^1^ Peking University China-Japan Friendship School of Clinical Medicine, Beijing, China; ^2^ Department of Cardiology, Heart Failure Center, China-Japan Friendship Hospital, Beijing, China

**Keywords:** heart failure with reduced ejection fraction, cardiac contractility modulation, guideline-directed medical therapy, cardiac remodeling, hypotension

## Abstract

**Background:**

Guideline-directed medical therapy (GDMT) for heart failure (HF) with reduced ejection fraction (HFrEF) has been demonstrated to significantly reduce morbidity and mortality. However, many patients, especially those with advanced HFrEF, are unable to tolerate optimal GDMT due to hypotension. Cardiac contractility modulation (CCM) is a novel therapeutic approach that enhances myocardial contractility and reverses cardiac remodeling, thereby improving cardiac function and quality of life in patients with HFrEF. However, whether CCM can bridge the hemodynamic vulnerability phase to facilitate GDMT optimization and improve patient prognosis remains unclear.

**Case presentation:**

A 56-year-old man with dilated cardiomyopathy and HFrEF (*N*YHA functional class III) had recurrent hospitalizations for HF over the past 4 years. Due to hypotension (systolic blood pressure ≤90 mmHg), the patient was unable to tolerate full-dose GDMT, with sacubitril-valsartan limited to 25 mg twice daily, metoprolol succinate to 23.75 mg once daily, and spironolactone to 20 mg once daily. After a comprehensive evaluation, a CCM device was implanted as the most effective and evidence-based option. Postoperatively, the patient's blood pressure gradually improved, allowing initiation of the four major therapeutic drug classes, which were uptitrated to the maximum tolerated doses. With regular follow-up for 12 months, the patient showed dramatic improvements in exercise capacity and quality of life. More surprisingly, there was significant improvement in cardiac structural and functional remodeling. Echocardiography revealed that left atrioventricular dimensions returned to normal, left ventricular ejection fraction (LVEF) increased from 15% to 48%, and left ventricular global longitudinal strain (GLS) improved from −3.3% to −16.2%. NT-proBNP levels also decreased from 6,553 pg/ml to within the normal range.

**Conclusion:**

This case suggests that CCM may serve as a promising strategy to address the issue of poor GDMT tolerance due to hypotension, thereby facilitating GDMT optimization and improving cardiac remodeling patients with HFrEF.

## 1 Introduction

Guideline-directed medical therapy (GDMT) has consistently demonstrated significant improvements in the prognosis of patients with heart failure (HF) with reduced ejection fraction (HFrEF). Despite robust evidence supporting its efficacy, real-world prescription and titration rates of GDMT remain suboptimal, particularly in patients with advanced or end-stage HF. Hypotension, occurring in approximately 70% cases, has been identified as the primary clinical barrier to the successful implementation of GDMT in HFrEF (1). Therefore, identifying strategies to ameliorate hypotension and improve tolerance to GDMT in these patients remain critical and unmet needs in the management of advanced HFrEF.

Cardiac contractility modulation (CCM) has emerged as an innovative device-based intervention for patients with advanced HFrEF. This modality delivers non-excitatory electrical impulses during the absolute refractory period, augmenting intracellular calcium handling through phosphorylation of phospholamban and thereby enhancing myocardial contractility without increasing oxygen demand (2). Studies have demonstrated its efficacy in improving cardiac function, exercise tolerance, and quality of life in HFrEF patients. However, whether CCM can enhance GDMT tolerability in advanced HFrEF patients remains uncertain.

This case report not only demonstrates substantial clinical improvements in the patient following CCM therapy, but also provides pioneering evidence of enhanced tolerance to GDMT, thereby enabling incremental titration to the maximum tolerated doses. Through regular follow-up evaluations at 1, 3, 6, and 12 months, the patient exhibited significant improvements in clinical symptoms, cardiac remodeling, functional capacity, and quality of life. These findings suggest that CCM may offer a promising therapeutic strategy to address the challenge of GDMT intolerance due to hypotension, thereby facilitating GDMT optimization and promoting cardiac remodeling patients with HFrEF. This case provides critical clinical insights for the implementation of CCM in patients with advanced HFrEF who exhibit intolerance to GDMT.

## 2 Case presentation

### 2.1 Clinical presentation

A 56-year-old male was diagnosed with HFrEF in October 2019. Despite receiving standard medical therapy for HFrEF, he experienced recurrent hospitalizations due to heart failure exacerbations over the past 4 years, and within the past year he experienced two hospital admissions. The left ventricular ejection fraction (LVEF) was 21%. Notably, due to hypotension, the patient was unable to tolerate the maximum recommended doses of GDMT, with his treatment regimen limited to sacubitril/valsartan 25 mg twice daily, metoprolol succinate 12.5 mg twice daily, spironolactone 20 mg once daily. The heart failure specialists at an external institution had recommended implantable cardioverter-defibrillator (ICD) placement. However, the patient explicitly declined the procedure, expressing a preference for ongoing pharmacological treatment. Additionally, he was prescribed furosemide 20 mg once daily for diuresis. Despite these interventions, the patient continued to suffer from exertional dyspnea and was unable to walk more than 300 m, without experiencing paroxysmal nocturnal dyspnea or chest pain. Consequently, he was referred to our institution for further management in October 2023.

The patient denied any recent a prodromal infection. He had a 4-year history of hyperlipidemia and had been taking rosuvastatin 10 mg daily. He denied a history of hypertension and diabetes. He reported a past history of smoking and alcohol consumption but had ceased both habits 1 year prior to admission. His alcohol use had lasted for 3 years, with an average daily intake of 10 g.

Upon admission, vital signs included a heart rate of 101 beats per minute, blood pressure of 90/71 mmHg, a respiratory rate of 21 breaths per minute, and a temperature of 36.1°C. His height was 160 cm, weight 55 kg, and BMI 21.5 kg/m^2^. Physical examination revealed bibasilar rales on lung auscultation and mild edema in both lower extremities. No cardiac murmurs were detected.

### 2.2 Laboratory and imaging examinations

Laboratory tests revealed elevated levels of N-terminal pro-B-type natriuretic peptide (NT-proBNP) at 6,553 pg/ml, troponin T at 0.025 ng/ml, alanine aminotransferase (ALT) at 81 IU/L, and aspartate aminotransferase (AST) at 70 IU/L. Serum creatinine was measured at 73.6 µmol/L, and the estimated glomerular filtration rate (eGFR) was calculated at 98.37 ml/min/1.73 m^2^.

**Figure 1:**
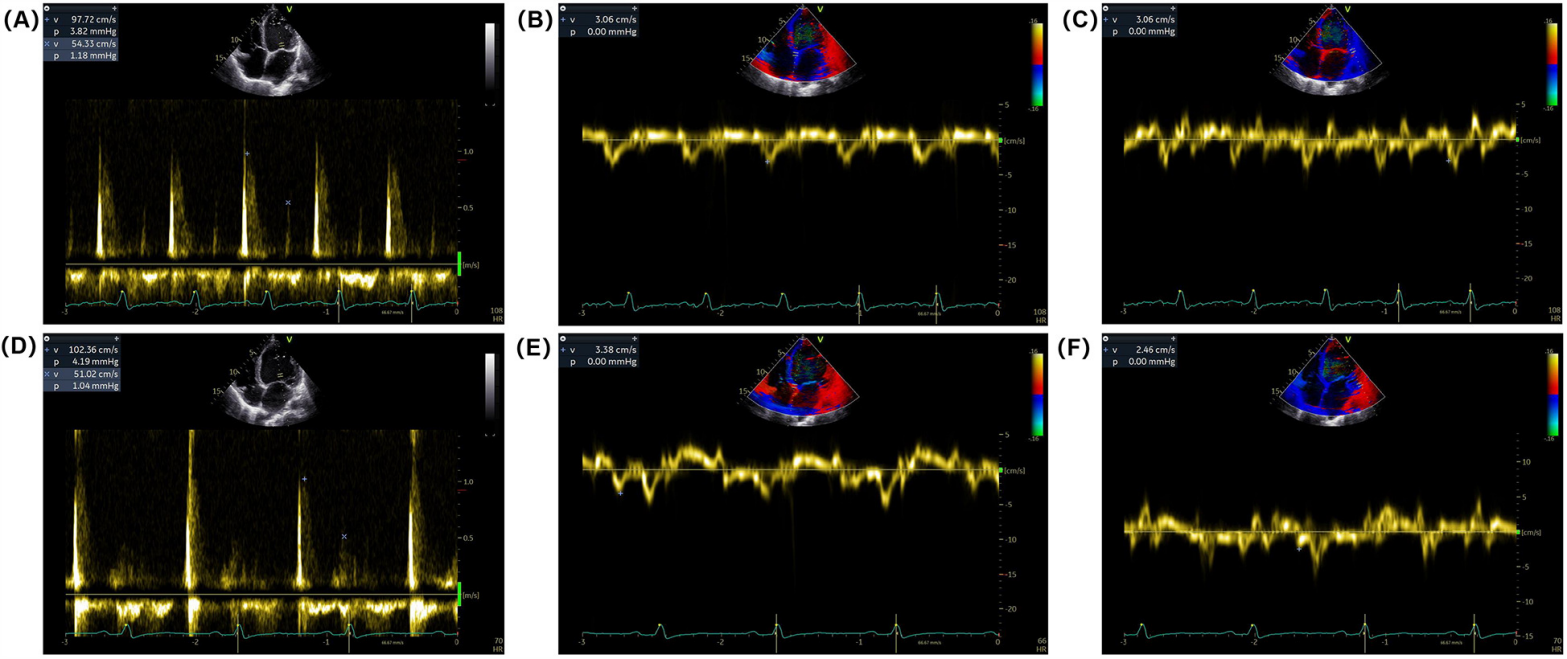
Mitral inflow and tissue Doppler imaging for diastolic function assessment. **(A)** Pulsed-wave Doppler recordings of mitral inflow showing **(E**,**A)** waves at admission. **(B,C)** Tissue Doppler imaging of the mitral annulus showing early diastolic velocity (e’) at the septal and lateral annulus at admission. **(D)** Pulsed-wave Doppler recordings of mitral inflow showing **(E**,**A)** waves at discharge. **(E**,**F)** Tissue Doppler imaging of the mitral annulus showing early diastolic velocity (e’) at the septal and/or lateral annulus at discharge.

**Figure 2:**
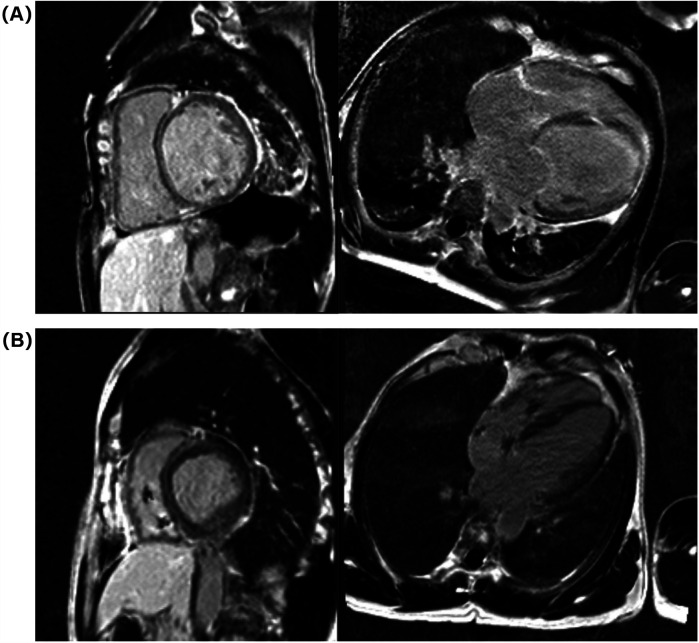
Cardiac MRI images at admission **(A)** and 1-year follow-up **(B)**.

Electrocardiogram (ECG) on admission revealed sinus rhythm with a heart rate of 101 beats per minute and a QRS duration of 118 ms. 24-h Holter monitoring revealed sinus rhythm (average heart rate 89 beats per minute), 44 premature atrial contractions, and two episodes of short-duration atrial tachycardia. Continuous electrocardiogram and blood pressure monitoring device also did not show any evidence of atrial fibrillation. Echocardiography demonstrated global cardiac dilatation with both impaired left ventricular systolic and diastolic dysfunction, along with reduced right ventricular systolic. The LVEF was only 15% with an E/e’ ratio of 32.3, mtral inflow and tissue doppler imaging for diastolic function assessment are shown in Figures 1A–C. With moderate mitral regurgitation. Left ventricular wall motion was globally impaired. LV global longitudinal strain (GLS) was −3.3%. Although the patient was diagnosed with dilated cardiomyopathy (DCM), definitive supporting documentation was unavailable. Ancillary tests were performed to investigate the etiology of HF, with coronary angiography revealing no significant coronary artery abnormalities. Cardiac magnetic resonance imaging (MRI), both plain and contrast-enhanced, showed end-diastolic volume (EDV) of 288 ml, end-systolic volume (ESV) of 217 ml, stroke volume (SV) of 71 ml. MRI further revealed patchy fibrosis is observed in the mid-myocardial layer of the left ventricle and in the anterior and posterior walls at the junction of the left and right ventricles (Figure 2A). No fibrosis is noted in the interventricular septal myocardium, with the total fibrotic area comprising less than 50%. Based on these findings, the patient was diagnosed with DCM. Right heart catheterization (RHC) revealed a CO of 1.93 L/min, a mean pulmonary artery pressure (mPAP) of 42 mmHg, a pulmonary capillary wedge pressure (PCWP) of 30 mmHg, and a pulmonary vascular resistance of 6.21 WU.

Upon admission, the patient was classified as New York Heart Association (NYHA) functional class III. Following diuretic therapy, the patient's exercise capacity showed slight improvement, with a 6-minute walk test (6MWT) of 354 m. The Kansas City Cardiomyopathy Questionnaire Clinical Summary Score (KCCQ-CSS) was 31.

### 2.3 Treatment following admission

Prior to admission, the patient was prescribed only 20 mg of oral furosemide daily and presented with a state of volume overload. Physical examination revealed bibasilar rales on lung auscultation and mild edema in both lower extremities. We intensified diuretic therapy while concurrently monitoring the patient's daily body weight, net fluid balance, lung signs, and serial changes in hospital NT-proBNP levels (Table 1). At admission, the patient presented with a resting heart rate of 101 bpm. Treatment with metoprolol succinate 12.5 mg twice daily combined with ivabradine 5 mg twice daily was initiated to reduce heart rate.

**Table 1:** Changes following decongestion therapy during hospitalization.

Parameters	Admission	Day 3	Day 7^a^	Day 10	Day 14
Rales	√	×	×	×	×
Weight (Kg)	56.0	54.0	53.5	53.0	53.0
Net fluid balance (ml)	–	−1,000	−500	−200	−100
NT-proBNP (pg/ml)	6,553	4,079	2,437	1,436	1,564
Diuretics	Furosemide 20 mg once daily (oral) + torasemide 10 mg once daily (intravenous)	Furosemide 20 mg once daily (oral) + torasemide 10 mg once daily (intravenous)	Furosemide 10 mg once daily (oral)	–	–

NT-proBNP, N-terminal pro-B-type natriuretic peptide. Day 7^a^: CCM treatment.

### 2.4 CCM treatment

During hospitalization, the patient's blood pressure (BP) fluctuated between 80–90/60–70 mmHg, and he was unable to tolerate GDMT. ECG revealed a QRS duration of less than 130 ms. Cardiac MRI showed less than 70% fibrosis in the interventricular septum, with no evidence of fibrosis at the apex. The patient had no history of myocardial infarction in the past 3 months and no episodes of angina in the past month. Based on these findings, the implantation of CCM device was considered as a strategy to improve the patient's cardiac function and prognosis. A pre-implantation levosimendan trial was conducted to evaluate the patient's responsiveness to CCM therapy. Intravenous levosimendan was administered at a dose of 0.10 µg/kg/min for a total duration of 5 h. Repeat echocardiography demonstrated an increase in LVEF from 22% to 30% (>5%), indicating a positive response to CCM. On day 7 of hospitalization, the patient underwent successful implantation of the CCM device. The Medtronic5076 two ventricular electrodes were placed via the right axillary vein, positioned at the high and low interventricular septal sites, with an inter-electrode distance of 41.82 mm. The electrodes were connected to the Optimizer Smart® pulse generator (Impulse Dynamics Inc., Orangeburg, NY, USA) and programmed to deliver electrical pulses at an initial voltage of 6.5 mV with a pulse width of 20 ms. The daily stimulation duration was set to 7 h, with a stimulation pattern of 1 h of stimulation followed by 3.4 h of rest, evenly distributed across a 24-h period.

One week post-surgery, the incision site had healed without complications, and no surgical adverse events were reported, including pain on stimulation, tricuspid valve insufficiency, or stimulation-induced arrhythmia. The patient's dyspnea gradually improved, and his functional status was reclassified as NYHA functional class II at discharge. BP increased to 95–105/70 mmHg, and NT-proBNP levels decreased to 1,564 pg/ml. Follow-up echocardiography demonstrated an improvement in LVEF to 40%, with an E/e’ ratio of 35.1 (Figures 1D–F) and mild mitral regurgitation. LVGLS improved to −6.4%.

### 2.5 Medical therapy and follow-up

Postoperatively, BP increased to 95–105/70 mmHg. The GDMT regimen was adjusted. GDMT was adjusted by up-titrating metoprolol succinate and discontinuing ivabradine. The discharge medication regimen included sacubitril/valsartan 25 mg twice daily, metoprolol succinate 59.375 mg once daily, empagliflozin 5 mg once daily, and spironolactone 20 mg once daily.

**Figure 3:**
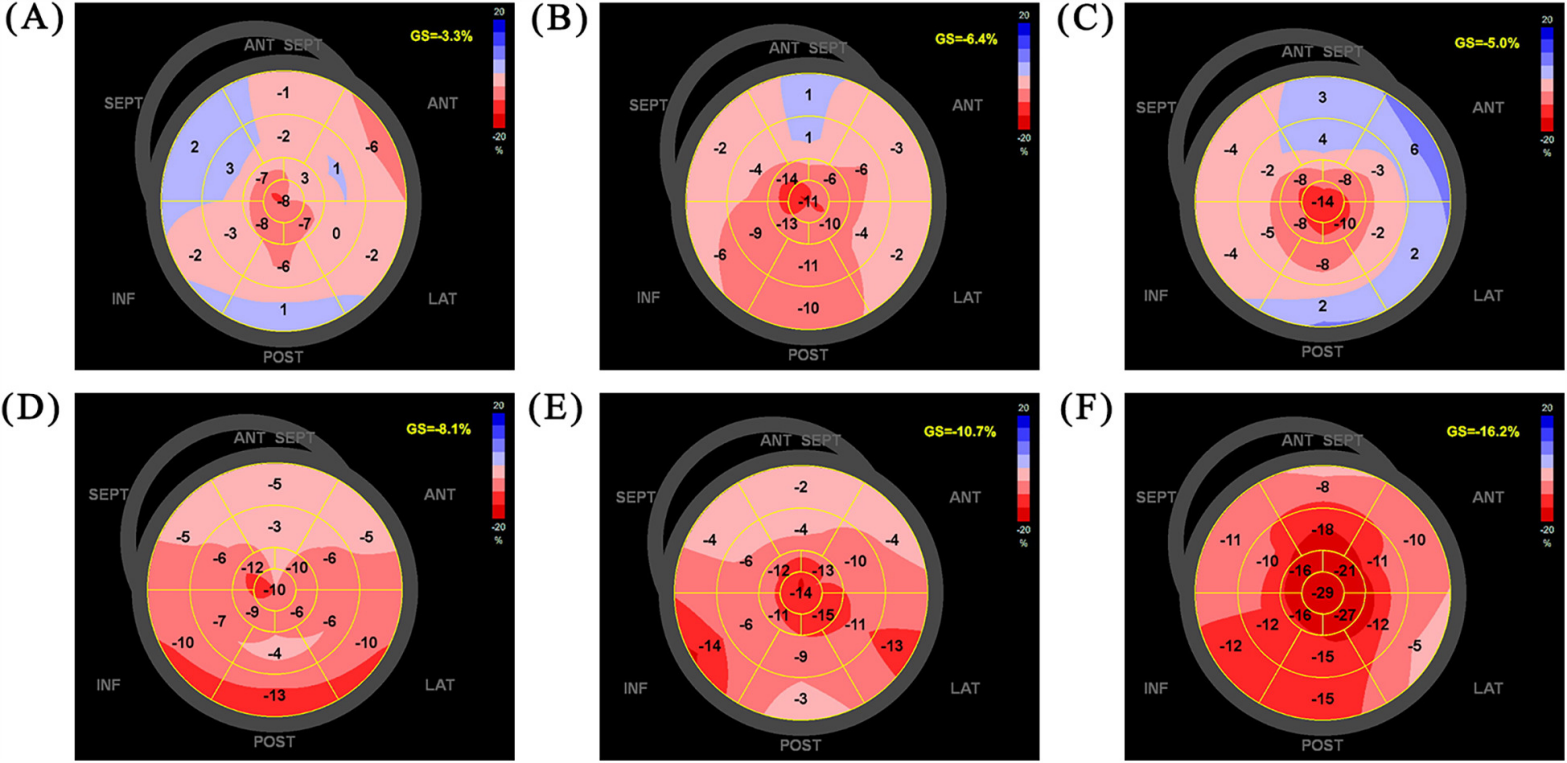
LVGLS on admission **(A)**, postoperative day 7 **(B)**, follow-up 1 m **(C)**, follow-up 3 m **(D)**, follow-up 6 m **(E)**, follow-up 12 m **(F)** LVGLS, left ventricular global longitudinal strain.

Follow-up evaluations were conducted at 1, 3, 6, and 12 months post-surgery, with the patient reporting no symptoms during these visits. BP remained stable, ranging from 105–110/70–80 mmHg. Clinical assessments demonstrated significant improvements in both exercise capacity and quality of life, as shown in Table 2. Follow-up echocardiography (LVGLS) revealed notable improvements in cardiac function (Figure 3). However, at 1 month follow up the LVEF was 34%. The CCM was checked to be working well and was not faulty. The patient had stopped taking some medications on his own 2 weeks after the operation as he had run out of empagliflozin and metoprolol succinate, and that he had stopped taking them for 2 weeks by the time of the 1-month follow-up.

**Table 2:** Clinical parameters at baseline and follow-up.

Parameters	Admission	Postoperative (Day 7)	Follow-up (1 m)	Follow-up (3 m)	Follow-up (6 m)	Follow-up (12 m)
Biomarker						
NT-proBNP (pg/ml)	6,553	1,564	1,332	855	293	91
Echocardiography						
LA (mm)	46	44	42	41	38	28
LVEDD (mm)	71	64	66	70	68	56
RA (mm)	49	37	39	33	32	27
RV (mm)	51	41	39	26	26	25
LVEF (%)	15	40	34	37	38	48
E/e’	32.3	35.1	27.3	17.7	14.6	9.3
TDI-s’ (cm/s)	8.92	12.1	9.5	9.1	9.4	9.3
TAPSE (mm)	13	20	16	15	16	17
LVGLS (%)	−3.3	−6.4	−5.0	−8.1	−10.7	−16.2
CMR						
ESV (ml)	217	–	–	–	–	63
EDV (ml)	288	–	–	–	–	126
SV (ml)	71	–	–	–	–	64
Exercise capacity						
6MWT (m)	354^a^	-	405	408	458	451
Quality of life						
KCCQ-CSS (scores)	31	-	88	89	90	92

NT-proBNP, N-terminal pro-B-type natriuretic peptide; LA, left atrium; LVEDD, left ventricular end-diastolic diameter; RA, right atrium; RV, right ventricular; LVEF, left ventricular ejection fraction; TDI, tissue Doppler imaging; TAPSE, tricuspid annular plane systolic excursion; GLS, global longitudinal strain; CMR, cardiac magnetic resonance; ESV, end-systolic volume; EDV, end-diastolic volume; SV, stroke volume; CO, cardiac output; 6MWT, 6-minute walk test; KCCQ-CSS, Kansas City cardiomyopathy questionnaire clinical summary score.

^a^ The patient's exercise capacity following diuretic therapy.

At the 1-month follow-up, metoprolol succinate has been titrated to 59.275 mg once daily. However, at 3-month follow-up, the patient's heart rate dropped to 50–55 bpm with mild fatigue, and then metoprolol succinate was downgraded to 47.5 mg once daily, with a return of the heart rate to 70 bpm and resolution of fatigue symptom.

**Figure 4:**
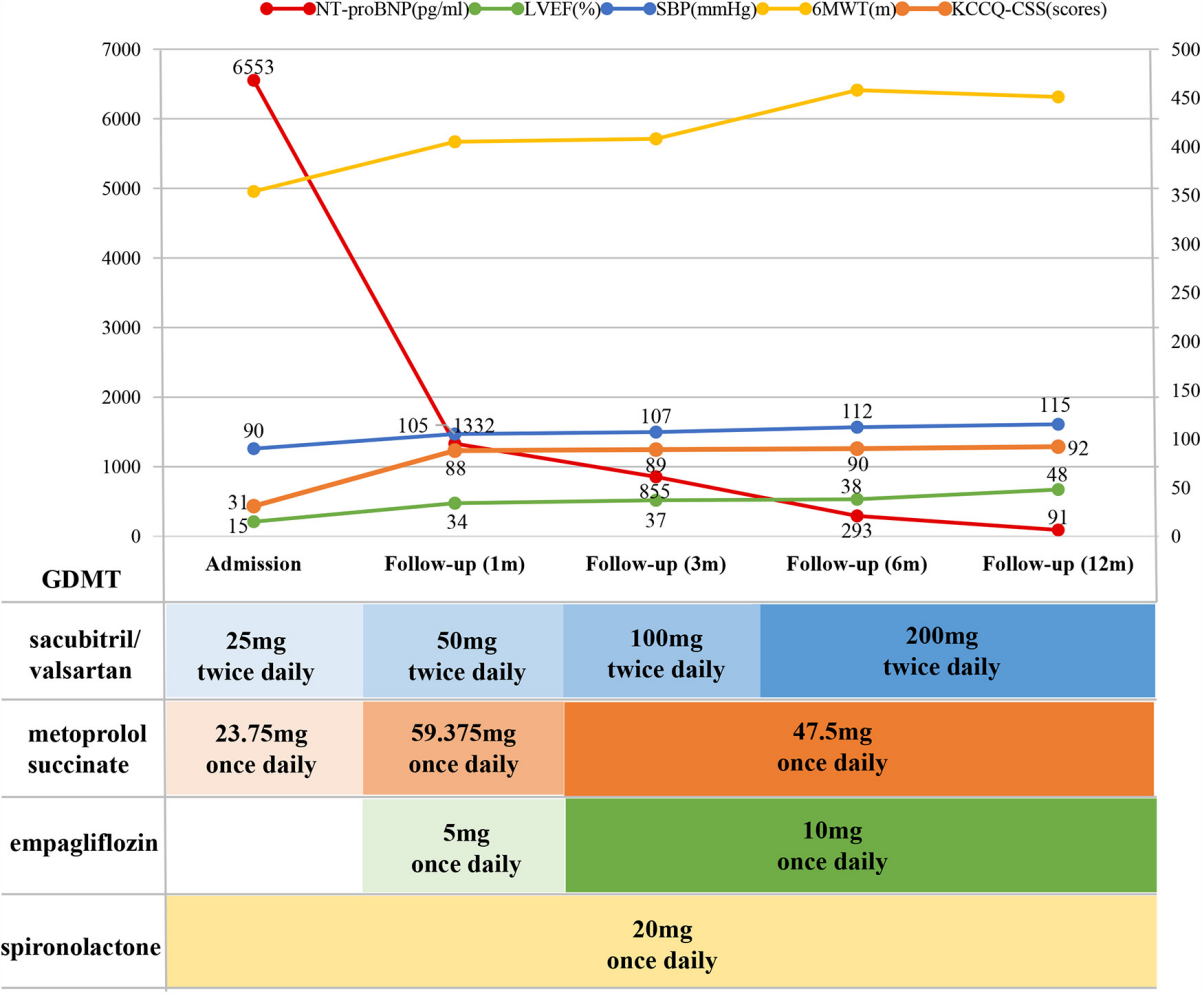
The GDMT optimization protocol and corresponding parameter adjustments during follow-up. NT-proBNP, N-terminal pro-B-type natriuretic peptide; LVEF, left ventricular ejection fraction; SBP, systolic blood pressure; 6MWT, 6-minute walk test; KCCQ-CSS, Kansas City cardiomyopathy questionnaire clinical summary score; GDMT, guideline-directed medical therapy.

At the 6-month follow-up, GDMT was titrated as follows: sacubitril/valsartan 200 mg twice daily, metoprolol succinate 47.5 mg once daily, empagliflozin 10 mg once daily, and spironolactone 20 mg once daily. A 12-month follow-up cardiac MRI showed no significant changes in the location or extent of fibrosis compared to pre-treatment findings (Figure 2B). The GDMT optimization protocol and corresponding parameter adjustments during follow-up were shown in Figure 4.

## 3 Discussion

This case report describes a patient with advanced HFrEF who was unable to tolerate GDMT. The implantation of CCM device resulted in significant improvements in cardiac function and provided the opportunity to optimize GDMT, allowing for titration to higher tolerated doses. The combined approach of GDMT and device-based treatment effectively improved exercise tolerance and quality of life in this patient. While previous studies have predominantly focused on the symptomatic benefits of CCM, this case uniquely demonstrates the potential of CCM in optimizing GDMT in advanced HFrEF.

Decongestion therapy is critical for patients with advanced HFrEF presenting with volume overload. During treatment, monitoring changes in volume status, including body weight, clinical signs, urine output/net fluid balance, and NT-proBNP levels, is essential. We initially intensified diuretic therapy to reduce volume overload. However, the patient's long-term intolerance to GDMT due to hypotension prevented further optimization. Hemodynamic assessment through RHC revealed severe cardiac dysfunction with a CO of 1.93 L/min, a mPAP of 42 mmHg, and a PCWP of 30 mmHg. Despite various adjustments in medication over the past 4 years, the patient's hemodynamic instability and intolerance to GDMT persisted.

We also considered the use of digoxin or levosimendan to enhance myocardial contractility and potentially improve blood pressure. However, studies (3) in chronic HFrEF patients have shown that while digoxin can increase LVEF, cardiac output, and reduce PCWP, it does not lead to an improvement in blood pressure. Furthermore, digoxin has been independently associated with an increased risk of all-cause mortality and all-cause readmission in patients with HFrEF (4, 5). Levosimendan, with its positive inotropic and vasodilatory effects, has been shown to reduce blood pressure within 24–48 h of administration, with systolic blood pressure decreasing by approximately 4 mmHg (6, 7). Thus, while both agents may improve symptoms and LVEF, they do not have a significant impact on blood pressure or long-term prognosis. In the present case, after the initiation of intensified diuretic therapy, the patient's symptoms were alleviated, although hypotension was observed. In such scenarios, device-based therapies should be considered.

According to heart failure guidelines (8, 9), for patients with chronic HFrEF exhibiting a LVEF ≤35% and persistent heart failure symptoms despite optimized GDMT for ≥3 months, clinicians should consider device-based therapies, including ICD and cardiac resynchronization therapy (CRT). In this case, ECG demonstrated no ventricular arrhythmias, absence of left bundle branch block, and revealed narrow QRS complexes (QRS duration <130 ms). Consequently, ICD should be considered. Over the past 4 years of treatment, heart failure specialists recommended ICD implantation to prevent malignant arrhythmic events; however, the patient repeatedly declined ICD implantation, opting to continue pharmacological therapy. And for patients with severely reduced LVEF and refractory symptoms, left ventricular assist device (LVAD) implantation (10) and cardiac transplantation are conventional options. While LVAD provides mechanical circulatory support, it carries significant risks of surgical complications, such as infection, bleeding, and thrombosis, and necessitates complex postoperative management. Cardiac transplantation remains limited by donor scarcity and potential immune rejection. As a minimally invasive intervention with a favorable safety profile and low complication rates, CCM effectively improved cardiac function without imposing the substantial risks associated with LVAD implantation or heart transplantation, such as device-related complications or immune rejection.

CCM is an emerging deviced-based therapy for chronic HF. The primary mechanism of CCM involves the application of biphasic electrical stimulation to the ventricular endocardium during the absolute refractory period. This stimulation prolongs the plateau phase of the myocardial action potential, leading to enhanced calcium (Ca^2+^) influx into the cells, enhancing the activity of the sarcoplasmic reticulum calcium ATPase, augmenting myocardial contractility, and promoting ventricular remodeling (2, 11), without an increase in oxygen demand of the myocardium. Previous clinical studies on CCM have demonstrated its efficacy in improving symptoms, exercise tolerance, and quality of life, as well as reduce hospitalizations for HFrEF in patients with NYHA functional class III or IV and narrow QRS complexes (QRS duration <130 ms) (12–15). However, the evidence remains limited, with a lack of high-quality randomized controlled trials, and the impact of CCM on all-cause mortality and long-term prognosis remains unclear. According to the HF management guidelines, CCM is considered for specific patient populations (LVEF 25%–45% and QRS duration <130 ms) as an adjunctive therapy following optimization of pharmacological treatment (8, 9). Although the benefits of CCM therapy have been more frequently reported in patients with an LVEF between 25% and 45%, emerging evidence suggests that CCM may also offer therapeutic benefits in patients with LVEF <25% (2, 12, 14, 16, 17), showing measurable improvements in ventricular function and quality of life. Due to concerns about surgical risks and costs, the patient declined both heart transplant and LVAD surgery, and instead accepted CCM implantation after a comprehensive assessment indicated potential benefit. The remarkable structural and functional cardiac remodelling observed in this case highlights the potential applicability of CCM in patients with LVEF <25%. However, further randomized controlled trials are warranted to confirm the therapeutic role of CCM in this population. On the 7th day after CCM therapy, the patient's LVEF had increased to 40%. Therefore, in HFrEF patients without malignant ventricular arrhythmias, CCM therapy may reduce the need for ICD implantation. This warrants further investigation in future studies.

GDMT remains the cornerstone of HFrEF management, improving symptoms, exercise capacity, and facilitating dramatic reverse remodeling (18–22). Guidelines of HF management recommend assessing eligibility for CRT+/−D in patients with persistent symptoms (NYHA class functional II–IV) and LVEF ≤35% without an evidence of LV dyssynchrony after at least 3 months of optimized GDMT (8, 9). GDMT typically includes renin-angiotensin-aldosterone system (RAAS) inhibitors, beta-blockers, sodium-glucose co-transporter 2 (SGLT2) inhibitors, and mineralocorticoid receptor antagonists (MRAs). Guidelines emphasize the importance of initiating GDMT with systematic tolerance evaluation and gradual dose titration toward either evidence-based target ranges or the maximal tolerated dose. Notably, hypotension remains as the primary limitation to the optimization of GDMT in advanced or end-stage HFrEF, especially beta blocker and RASi (1). A study demonstrated that hypotension prevents the use of beta-blockers in approximately 30% of patients and delays the initiation of RAAS inhibitors in 23% of cases (23). However, CCM improves hemodynamics and thus facilitates the optimization of GDMT in previously intolerant patients (24). Mechanistically, CCM enhances myocardial contractility through calcium-mediated electromechanical coupling, without elevating myocardial oxygen demand (25–28), thereby improving cardiac output and peripheral perfusion. This stabilization of BP enables the optimization of GDMT in hypotensive, advanced HFrEF patients who were previously intolerant to GDMT.

Although this case demonstrates significant benefits from CCM therapy, careful preoperative assessment is crucial to determine the potential benefits. This includes evaluating CCM responsiveness through the levosimendan test and assessing myocardial fibrosis burden and location with cardiac MRI. Prior to CCM implantation, it was essential to assess the expected response (11, 29). Preoperative evaluation of fibrosis burden and location by cardiac MRI played a crucial role in assessing the potential benefit of CCM therapy. First, extensive myocardial fibrosis generally indicates poor responsiveness to ventricular reverse remodeling. Second, CCM electrical stimulation is ineffective in areas with myocardial fibrosis. Implanting electrodes in fibrotic regions would be unlikely to improve local or global myocardial function and could increase the risk of septal perforation. Therefore, patients with significant interventricular septal fibrosis may experience a suboptimal response to CCM therapy. Moreover, patients who have previously responded well to levosimendan typically exhibit favorable responsiveness to CCM. For heart failure patients, cardiopulmonary exercise testing (CPET) is not only a key measure of functional assessment but also an important prognostic indicator (30, 31). We only conducted a 6MWT in this case, but in the future, we will implement CPET comprehensively in heart failure patients.

## 4 Conclusion

In this case report, we present a patient with advanced HFrEF who was unable to tolerate GDMT but exhibited improved tolerance to optimized GDMT following CCM therapy. The combination of GDMT and device-based treatment resulted in substantial improvements in cardiac function, exercise tolerance, and quality of life. Based on our clinical experience, we propose that for advanced HFrEF patients who are intolerant to GDMT, early consideration of CCM therapy, prior to more advanced interventions, may be beneficial. A comprehensive evaluation and regular follow-up, in conjunction with the optimization of GDMT, could potentially lead to improved long-term outcomes for these patients.

## Data Availability

The original contributions presented in the study are included in the article/Supplementary Material, further inquiries can be directed to the corresponding author.
