# Supplementary material for: Cardiac contractility modulation to enhance optimized medical therapy and improve cardiac remodeling in advanced heart failure: a case report
Source: Front Cardiovasc Med. 2025 Jun 6;12:1577680. doi: 10.3389/fcvm.2025.1577680 (PMC12179112; doi:10.3389/fcvm.2025.1577680)
Supplement: Supplementary file 1 [file Datasheet1.docx]

Supplementary Material

# Supplementary Figures


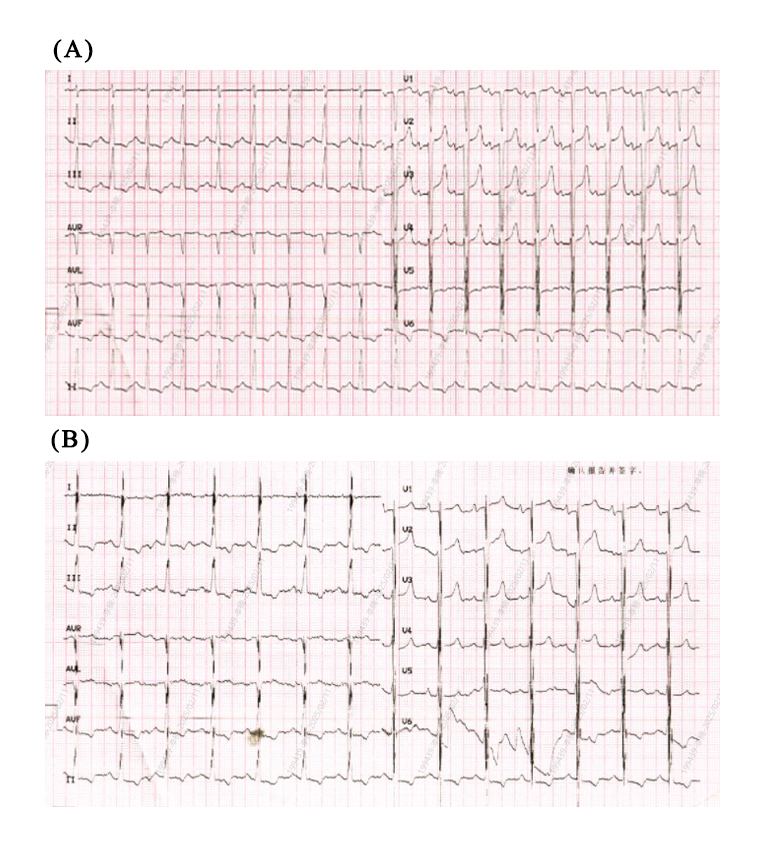


**Supplementary Figure 1.** Preoperative (A) and postoperative (B) ECGs of CCM therapy.


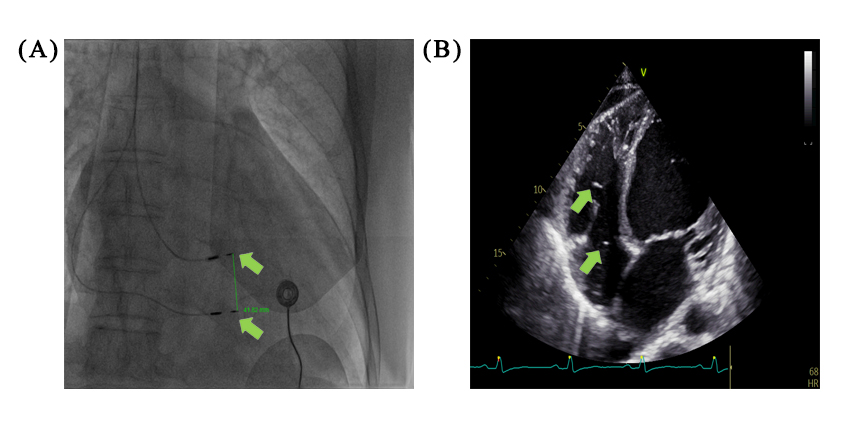


**Supplementary Figure 2.** Postoperative chest X-ray (A) and echocardiography (B) showed that the positions of the two CCM electrodes were optimal.
